# Synthetic Marijuana: Assessment of Usage, Motivation and Associated Risks in Adolescent Substance Users

**DOI:** 10.1177/29768357241254258

**Published:** 2024-05-17

**Authors:** Raman Baweja, Sara Mills-Huffnagle, Amanda Jernigan, Nungshitombi Chongtham, Daniel Waschbusch, James G Waxmonsky

**Affiliations:** 1 Pennsylvania State College of Medicine, Hershey, PA, USA; 2 Gaudenzia, Inc., Harrisburg, PA, USA

**Keywords:** Synthetic marijuana, natural marijuana, benefits, risks, adolescent

## Abstract

**Objectives:**

Use of Synthetic and designer products, including synthetic marijuana (SM), among adolescents poses a major risk to public health. Little is known about the motivating factors of synthetic substance use in adolescents. This study examined the motivations, predictors, perceived risks and benefits, and differences with SM versus natural marijuana among adolescents.

**Methods:**

Between April 2016 and May 2018, a convenience sample of adolescents receiving substance use treatment from a local counseling center completed an anonymous survey to assess the use of natural and synthetic marijuana use, the Strengths and Difficulties Questionnaire to assess levels of current psychiatric symptoms and the Community Assessment of Psychic Experiences-Positive Scale to assess symptoms of psychosis.

**Results:**

A total of 80 adolescents (age range of 14-18 years; 71% male; 53% Caucasian) completed the study. Of these, 39 (49%) reported natural marijuana use (natural marijuana users) and 41 (51%) reported both synthetic and natural marijuana use (dual users). The most commonly reported reasons for using SM were its low cost and reduced risk of detection. Participants who were familiar with SM and reported a desire to avoid detection on drug tests were likelier to use SM (all *P* < .05). Dual users reported more benefits and risks associated with SM use when compared to natural marijuana users (*P* ⩽ .05). The use of SM also heightened the perceived medical risks of natural marijuana, including seizures and respiratory issues (*P* < .05), compared with natural marijuana users. While dual users self-reported more conduct (*P* = .009) and externalizing problems (*P* = .024) when compared to natural marijuana users, there were no group differences in psychotic symptoms, nor correlations with the frequency of synthetic or natural marijuana use and psychotic symptoms except that persecutory ideation correlated with the frequency of natural marijuana use during the past 12 months (*r_p_* = 0.28, *P* = .04).

**Conclusions:**

These results suggest that reports of cost savings, and lack of detection on urine drug screens are common reasons for SM use in adolescents despite being aware of the risks of using SM. Perceived benefits of using synthetics and other novel substances appear diverse, and merit further exploration as a better understanding of what motivates adolescents to use specific novel substances may guide prevention and treatment efforts.

## Introduction

Synthetic marijuana (SM) consists of dried shredded plant material sprayed with various synthetic materials derived from laboratory-made cannabinoid receptor agonists.^1^ The first reports of SM use in the United States (US) occurred in 2009, followed by a dramatic spread in its use among adolescents and young adults.^2^ In 2013, the Monitoring the Future Study (MFS) found that 10% of high school seniors used SM in the last year, with 3% using it frequently.^3^ While the use of synthetic marijuana has declined in adolescents over the past decade, its use is still common; for example, the MFS recently reported a significant increase in SM use in high school seniors between 2021 and 2022.^4^ Moreover, the 2021 Youth Risk Behavior Survey reported a 6.5% prevalence in lifetime SM use among a representative group of US high school students.^5^ The poison control centers reported 984 calls for synthetic cannabinoid-related exposure cases in 2021 and 372 calls as of July 2023.^6^

Adverse effects of SM use are reported to be more severe and unpredictable than natural marijuana.^7^ For example, SM use in adolescents is associated with a much higher risk for depressive disorder, anxiety disorder, conduct disorder, and auditory hallucinations than natural marijuana use.^8^ Many of the synthetic derivatives found in SM are full agonists at the cannabinoid-1-receptor with higher binding affinity than tetrahydrocannabinol.^9^ The exact psychoactive ingredients in a single dose of SM can be appreciably variable, leading to highly fluctuating experiences within the same user. While the most commonly reported adverse event of SM use has primarily been agitation, severe adverse events have also been reported, with nearly 1 in 8 calls involving a potentially life-threatening event.^10^ SM can induce acute psychosis that may persist for up to a month. SM use has been associated with increased rates of persistent psychotic symptoms.^1,10^ This risk is much higher for younger SM users.^11^

Prevention efforts are ideally targeted toward the groups most likely to engage in use, emphasizing the importance of identifying risk factors for use. To date, in adolescents and young adults, the most established risk factor for SM use is the concurrent use of natural marijuana and other substances.^3,12-15^ In both adults and adolescents, the presence of depressive symptoms and the total burden of mental health symptoms predict SM use.^12,16^

Adult users often report SM as less palatable than natural marijuana and describe the latter as safer, more consistent, and a better value for the price, leading some to conclude that the desire to avoid detection on drug tests is a primary reason for SM use.^17^ Indeed, most synthetic cannabinoids cannot be detected on standard drug screens,^18^ and adults report that the difficulty of detecting SM use is a common reason for using it.^19^ SM products can be more potent and efficacious than natural marijuana, with a more intense and diverse array of psychoactive effects.^10,14^ However, perceived risks, benefits, and other risk factors for substance use vary by age,^20^ emphasizing the importance of assessing adolescent motivations for SM use.

Very little is known about the frequency of SM use and the motivating factors behind synthetic versus natural marijuana use in adolescents. Understanding these motivations may enhance prevention and intervention efforts. For instance, if adolescents place little value on the risk of adverse effects, efforts focused mainly on publicizing these risks will likely have limited impact. However, little is known about how adolescents perceive the risks and benefits of SM or their motivations for use. The aim of this study was to examine the motivations, predictors, perceived risks and benefits, and differences with SM versus natural marijuana among adolescents. To this end, we surveyed a group of adolescents from a local substance use treatment program at high risk for SM use. They completed an anonymous survey about their substance use, including their motivations for use, perceived risks and benefits of SM, and perceived differences versus natural marijuana.

## Methods

### Participants

A convenience sample of 80 adolescents between 14 and 18 years old who received substance use treatment at a community adolescent substance use treatment program were invited to participate in this study. Eligible participants were fluent in written and spoken English. Around two-thirds of adolescents enrolled in the program were court-ordered, with the rest referred from local schools (through a student assistance program) or by parents. Participation in this study was voluntary and verbal consent was given by the participant before study participation. Participants received a $10 gift card to complete the survey.

### Procedures

Between April 2016 and May 2018, treatment center staff engaged all program attendees about completing the anonymous online survey. Adolescents who consented to participate completed the online surveys using a tablet at the treatment center. Participants completed the novel Substance Abuse Survey that measured usage and perceived risks and benefits, the Strengths and Difficulties Questionnaire (SDQ),^21^ and the Community Assessment of Psychic Experiences-Positive Scale (CAPE-P15).^22^ Together, the surveys took about 20 minutes to complete. Responses to the survey were anonymous and stored on REDCap (Research Electronic Data Capture), a secure, web-based application designed for research studies. The local governing Institutional Review Board approved this study.

#### Assessments

##### Substance abuse survey

This measure was created for the study to detail the frequency of recent natural and SM use, reasons for use, and perceived risks and benefits. It included multiple-choice and open-ended questions (see attached Supplement). Since concurrent substance use is an established risk factor for synthetic marijuana use in adults,^19^ this survey also asked about the use of other illicit substances.

##### Strength and Difficulties Questionnaire (SDQ)

Participants completed the self-report version of SDQ^21^ to assess current psychiatric symptoms and their association with SM use. The SDQ consists of 25 items rated on a 3-point Likert scale (not true, somewhat true, and certainly true) to evaluate 5 subscales: *Emotional Symptoms, Conduct Problems, Hyperactivity-inattention, Peer Problems*, and *Prosocial Behaviors.* Higher scores represent more problems except for the *Prosocial Behaviors* subscale. The externalizing score ranges from 0 to 20 and is the sum of the *Conduct Problems* and *Hyperactivity-inattention* subscales. The internalizing score ranges from 0 to 20 and is the sum of the *Emotional Symptoms* and *Peer Problems* subscales. The total difficulties score ranges from 0 to 40 and is the sum of all the scales except the *Prosocial Behaviors* subscale.

##### Community Assessment of Psychic Experiences-Positive Scale (CAPE-P15)

The CAPE-P15 is a self-reported scale of lifetime psychotic-like experiences.^22^ The 15-item scale addresses the following domains: persecutory ideation, bizarre experiences, and perceptual abnormalities. The scale also assesses the frequency of psychotic-like experiences ranging from 1-never, 2-sometimes, 3-often, to 4-nearly always for frequency. Higher scores indicate a higher frequency of psychotic-like experiences.

### Analytic plan

Since all participants (N = 80) reported natural marijuana use, participants were divided into 2 groups: those who used natural marijuana only (natural marijuana users, n = 39) and those who used both natural and synthetic marijuana (dual users, n = 41). Descriptive statistics were computed to examine respondent characteristics, substance use patterns, and perceived risk and benefits. Due to missing data, the sample size for specific analyses involving the SDQ and CAPE-P15 was reduced to 27 and 32 for natural marijuana users and dual users, respectively. Chi-square/Fisher’s exact tests were computed for categorical variables and independent *t*-test for continuous variables to identify factors associated with SM usage. All *t*-tests were two-tailed with significance set at *P* ⩽ .05. Correlations between the CAPE-P15 scores, and frequency of natural and synthetic marijuana use were also evaluated, with significance set at *P* ⩽ .05.

## Results

### Sociodemographic

Participants were between the ages of 14 and 18 years old, with the majority identifying as male (71%) and Caucasian (53%; Table 1). The most common self-reported comorbid psychiatric diagnoses were attention-deficit/hyperactivity disorder (39%), mood disorders (including bipolar disorder, 27.5%), and anxiety disorders (19%). The average age of first natural marijuana use was 12.67 years (SD2.25). The preferred substance of choice was natural marijuana (74%), followed by alcohol (7.5%), with prescription opioids, alprazolam, and hallucinogens each reported as a preferred substance for 2.5% of the sample. Most (91%) participants had heard of SM. There were 41 (51%) participants who reported using SM versus 39 (49%) who reported only natural marijuana use. Among dual users, 39% (n = 16) used synthetic marijuana over the last year, and 12% (n = 5) reported using it over the last month. The remaining participants (n = 20) had used it more than a year ago. In comparison, use of natural marijuana was much higher, with 59% reporting using it over 40 times per year. Of the participants (n = 43) who had mandated drug tests a year before enrolling into the program, more than half (n = 25, 58.1%) reported concerns about their use being detected.

**Table 1.** Characteristics of NM and dual users.

	NM users (N39)	Dual user (N41)	*χ*2/*t*-test value (DF)	*P*-value
Heard about synthetic marijuana (%)	32 (82.1)	41 (100.0)	8.06 (1)	.005
Male (%)	27 (69.2)	30 (73.2)	1.29 (2)	.53
Age in years when started using NM, mean (SD)	12.60 (2.9)	12.73 (1.41)	−2.33 (76)	.82
Race (%)				
Caucasian	24 (61.5)	17 (41.5)	5.84 (5)	.32
African American	7 (17.9)	11 (26.8)		
Hispanic	1 (2.6)	4 (9.8)		
Mixed	6 (15.4)	6 (14.6)		
Other	0	2 (4.9)		
Did not answer	1 (2.6)	1 (2.4)		
Highest grade (%)				
7th grade	0	1 (2.4)	13.73 (15)	.55
8th grade	4 (9.8)	5 (12.2)		
9th grade	4 (9.8)	6 (14.6)		
10th grade	9 (23.1)	9 (21.9)		
11th grade	14 (36)	12 (29.3)		
12th grade	8 (20.5)	3 (7.3)		
GED	0	2 (4.9)		
Did not answer	0	3 (7.3)		
Personal income/week (%)				
None	14 (35.9)	20 (48.8)	8.52 (7)	.289
$1-49	5 (12.8)	2 (4.9)		
$50-100	6 (15.4)	5 (12.2)		
$101-200	8 (20.6)	5 (12.2)		
$>200	4 (10.3)	2 (4.9)		
Did not answer	2 (5.1)	7 (17.1)		
Nights with friends outside/week (%)				
None	9 (23.1)	13 (31.7)	2.27 (4)	.686
Once	5 (12.8)	7 (17.1)		
Two-three	14 (35.9)	13 (31.7)		
>four time	10 (25.6)	8 (19.5)		
Did not answer	1 (2.6)	0		
Friends use SM (%)				
None	28 (71.8)	19 (46.3)	5.85 (2)	.054
Just a few	9 (23.1)	20 (49.8)		
Most	0	0		
All	0	0		
Did not answer	2 (5.1)	2 (4.9)		
Friends use NM (%)				
None	1 (2.6)	1 (2.4)	5.48 (4)	.969
Just a few	9 (23.1)	7 (17.1)		
Most	21 (53.8)	25 (61.0)		
All	7 (17.9)	7 (17.1)		
Did not answer	1 (2.6)	1 (2.4)		
Friends use another drug (%)				
None	10 (25.6)	11 (26.8)	2.82 (4)	.59
Just a few	22 (56.4)	21 (51.2)		
Most	3 (7.7)	7 (17.1)		
All	1 (2.6)	0		
Did not answer	3 (7.7)	2 (4.9)		
Used NM in the past 12 mo				
None	2 (5.1)	3 (7.3)	9.18 (7)	.24
1-5 times	3 (7.7)	2 (4.9)		
6-9 times	3 (7.7)	1 (2.4)		
10-19 times	3 (7.7)	2 (4.9)		
20-39 times	1 (2.6)	4 (9.8)		
>40 times	24 (61.5)	23 (56.1)		
Did not answer	3 (7.7)	6 (14.6)		
Preferred substance of choice (%)				
Marijuana	25 (64.1)	32 (78.0)	30.39 (31)	.50
Alcohol	2 (5.1)	2 (4.9)		
Marijuana + Alcohol	1 (2.6)	1 (2.4)		
Others	6(15.4)	4 (9.8)		
None	5 (12.8	2 (2.4)		
Household (%)				
Both parents	12 (30.8)	13 (31.7)	4.76 (3)	.19
Single parent	25 (64.1)	22 (53.7)		
None	1 (2.6)	6 (14.6)		
Did not answer	1 (2.6)	0		
Parents highest level of schooling (%)				
<High school	5 (12.8)	8 (19.5)	8.05 (6)	.23
High/school/GED	8 (20.5)	13 (31.7)		
Attended college	3 (7.7)	5 (12.2)		
Graduated colleges	12 (30.8)	7 (17.1)		
Professional college	7 (17.9)	2 (4.9)		
Did not answer	4 (10.2)	6 (14.6)		
Have job (%)	17 (43.6)	12 (29.3)	1.77 (1)	.183
School enrollment (%)	35 (89.7)	34 (82.9)	2.37 (2)	.28
Missed school days in the last 3 mo for any reason, mean (SD)	7.56 (11.21)	9.85 (17.15)	−0.65 (65)	.519
Missed school days in the last 3 mo for D&A use, mean (SD)	2.51 (9.09)	5.54 (16.4)	−0.95 (66)	.346
Do you have drug test before starting treatment	20 (51.3)	23 (56.1)	0.19 (1)	.418
If had drug test—How worried about failing it (%)	11 (28.2)	14 (34.1)	1.67 (5)	.89
Reason to use SM—to avoid detection on drug test (%)	2 (5.1%)	12 (29.3)	8.07 (1)	.007
History of arrest (%)	27 (69.2)	31 (75.6)	0.43 (2)	.81
Mental health diagnosis (%)	19 (48.7)	24 (58.5)	2.79 (3)	.425
Pharmacological treatment for mental health diagnosis (%)	15 (38.5)	20 (48.8)	3.25 (2)	.197
Hospitalization for mental health diagnosis (%)	3 (7.7)	8 (19.5)	5.77 (3)	.123
Family History of D&A use (%)	14 (35.9)	21 (51.2)	4.61 (3)	.203

Abbreviations: D&A, drug and alcohol; DF, degrees of freedom; NM, natural marijuana; SD, standard deviation.

Among dual users, the majority (98%, n = 40) used SM by smoking, and 8 participants (20%) required hospitalization or medical treatment related to synthetic marijuana use. The most commonly reported reasons for SM use among dual users included cheaper cost versus alternatives (34%), avoiding being detected on drug tests (29%), the perceived high (22%), and ease of access (20%).

Dual users, compared to those who used only natural marijuana, heard more about SM (*χ*2 = 8.06, *P* = .005), and were more likely to voice a desire to avoid detection on drug tests (*χ2* = 8.07, *P* = .007). Although not statistically significant at the .05 level, the trend suggests that dual users might have more friends who use SM (*χ2* = 5.82, *P* = .054). There was no difference for other sociodemographic factors, mental health histories, legal issues, educational achievement, preferred substance of choice and frequency of natural marijuana use among natural marijuana users and dual users.

## Perception About Benefits and Risks

Dual users reported more benefits of using SM versus those who had never used synthetic marijuana (high quality, onset, and duration of high, and value for money; all *P*’s ⩽ .05; Table 2). In contrast, the benefits of using natural marijuana were not rated more positively by dual versus natural marijuana users. Dual users also reported more risks with SM use versus adolescents who had never used it (less safe, addiction risk, hangover effects, seizure risk, cardiac problems, paranoia, aggression, breathing problems, and risk of mood swings; all *P*’s ⩽ .05). Interestingly, using SM was associated with heightened perceived medical risks [risk of seizures (*t* = −3.08, *P* = .003) and breathing problems (*t* = −2.62, *P* = .01)] with natural marijuana.

**Table 2.** Perception about NM and SM among NM versus dual users.

	About SM				About NM			
	NM users (39)	Dual users (41)	*T*-test value (DF)	*P*-value	NM users (39)	Dual users (41)	*T*-test value (DF)	*P*-value
Experience								
How good the High is (1 very poor, 10 great)	0.74	4.38	−5.50 (69)	<.001	7.14	7.30	−0.235 (75)	.81
How long the High lasts (1very short, 10 very long)	0.60	3.69	−7.45 (67)	<.001	4.92	5.93	−1.56 (75)	.12
How fast it takes to feel effects (1 very slow, 10 very fast)	0.73	5.73	−8.74 (68)	<.001	5.25	5.60	−0.54 (74)	.59
Value for my money (1 terrible, 10 great value)	1.63	4.51	−3.73 (69)	<.001	4.78	5.20	−0.57 (75)	.57
Risk								
Safest (1 very safe, 10 very dangerous)	1.97	6.59	−6.44(69)	<.001	2.51	3.2	−0.87 (75)	.39
Hangover effects (1 none, 10 severe)	0.93	2.78	−2.81(69)	.006	1.08	1.63	−1.10 (75)	.28
Risk of (1 no risk, 10 big risk)								
Seizures	1.33	4.24	−3.90 (69)	<.001	0.54	1.5	−1.83 (75)	.07
Heart problems	1.87	4.17	−3.05 (69)	.003	0.38	1.95	−3.08 (75)	.003
Feeling paranoid	1.8	5.8	−5.48 (69)	<.001	2.19	3.3	−1.63 (75)	.11
Becoming aggressive	1.53	4.15	−3.71 (69)	<.001	1.16	1.9	−1.31 (75)	.19
Breathing problems	1.57	4.18	−3.56 (67)	.001	1	2.63	−2.62 (72)	.01
Mood swings	1.45	4.43	3.99 (67)	<.001	1.57	2.29	−1.17 (73)	.25
Addiction risk	1.6	4.44	−3.79 (69)	<.001	1.76	2.59	−1.49 (74)	.14

Abbreviations: DF, degrees of freedom; NM, natural marijuana; SM, synthetic marijuana.

## Psychiatric Comorbidity

While mental health diagnoses and treatment rates were higher in dual users, none of these differences reached significance (see Table 1). Dual users self-reported more conduct (*t* = −2.72, *P* = .009) and externalizing problems (*t* = −2.32, *P* = .024) and trended toward a higher total score on the SDQ when compared to natural marijuana users (*t* = 1.92, *P* = .06; Table 3). While several items on the CAPE-P15 (paranoid ideation, bizarre experiences, and perceptual anomalies) and the total CAPE-P15 score were higher in the dual users versus natural marijuana users, none of these differences reached significance (Table 3). There were no significant correlations with the frequency of synthetic or natural marijuana use in the last month and any items on the CAPE-P15, except for persecutory ideation, which significantly correlated with the frequency of natural marijuana use in the past 12 months (*r_p_* = 0.28, *P* = .04).

**Table 3.** Comparison of psychiatric comorbidity among NM versus dual users.

Score	NM users (N27)	Dual users (32)	*t*-test value (DF)	*P*-value
	Mean (SD)	Mean (SD)		
SDQ				
Emotional problem	3.26 (2.67)	3.56 (2.29)	−0.47 (57)	.64
Peer problem	2.52 (1.72)	2.81 (1.51)	−0.699 (57)	.49
Internalizing scale	5.78 (3.26)	6.38 (3.11)	−0.719 (57)	.48
Hyperactivity	4.7 (2.55)	5.34 (2.04)	−1.07 (57)	.29
Conduct problem	2.48 (1.87)	3.81 (1.87)	−2.72 (57)	.009
Externalizing scale	7.19 (3.49)	9.16 (3.05)	−2.32 (57)	.024
Total score	12.96 (5.83)	15.53 (4.44)	−1.92 (57)	.06
Prosocial	7 (2.3)	6.84 (2.1)	−0.273 (57)	.79
CAPE-P15				
Paranoid ideation	3.63 (3.51)	3.66 (2.59)	−0.047 (58)	.96
Bizarre experiences	1.89 (2.5)	2.85 (3.6)	−1.17 (58)	.25
Perceptual anomalies	0.48 (0.89)	1.09 (1.76)	−1.94 (58)	.11
Total score	6 (6.01)	7.6 (6.66)	−0.976 (58)	.39

Abbreviations: CAPE-P15, Community Assessment of Psychic Experiences-Positive Scale; DF, degrees of freedom; NM, natural marijuana; SDQ, strength and difficulties questionnaire.

## Discussion

About half of the participants between the ages of 14 and 18 treated for substance use disorders at a community adolescent substance use treatment program reported using SM. All SM users also used natural marijuana, most using over 40 times per year. Adolescents were more likely to use SM if they reported being familiar with SM, and believed that the drug was undetectable on urine drug screens. Income, educational status, other demographic variables, and past psychiatric history measures were not associated with SM use. In contrast, higher self-reported externalizing symptoms were associated with SM use. The latter is an established risk factor for adolescent substance experimentation, misuse, and disorder.^23^

In this sample, natural marijuana was the most common substance reported. All dual users reported using natural marijuana, with nearly two-thirds using it 3 times a month or more. However, the amount of overall substance use was not appreciably different between dual users and natural marijuana users, suggesting that the frequency of use does not reliably predict who may transition to novel substances, including synthetic substances. These results indicate that it is challenging to identify the adolescent active substance users who are most likely to progress to the use of novel, more dangerous substances based solely on information typically recorded in the medical record. It may prove more beneficial to assess their social networks and personal motivations for use than to review demographic or treatment factors when trying to determine their risk.

Peer influence plays a crucial role in initiating and continuing substance use.^24^ Adolescents who were familiar with SM or had friends who used it were likelier to use these products. Past work also reported that adolescent marijuana use is more highly associated with best friend marijuana use than with older sibling or adult (most important figure in their life) marijuana use.^25^ In addition, synthetic users had higher rates of self-reported conduct and other externalizing symptoms. Others have also reported that externalizing symptoms at an early age (eg, age 11) are a robust predictor of early-onset substance use, including alcohol, nicotine, and cannabis.^23^

These results suggest that motivating factors of SM use in adolescents include lower cost, lack of detectability on urine drug screens, psychoactive effects of the drug (eg, a better high), and ease of access. Past work has also reported that adolescents and young adults often use the least expensive and the most accessible synthetic products.^19,26^ In our study, participants were more aware that SM was undetectable on urine drug screens. These results are similar to what has been previously reported by young adults in a large global sample,^17^ and reduced risk of drug detection has also been reported as a common motivating factor for SM use in adults.^19^ Data on the importance of evading detection are less consistent in adolescents versus adults,^17,19^ possibly because of the reduced work obligations, legal challenges, and financial responsibilities of younger users. However, in our study of adolescents currently in treatment for a substance use disorder, the risk of detection was a motivating factor, possibly because many already had experienced drug-related legal repercussions. These motivations suggest that there will be a continual appeal to inexpensive novel agents that are challenging to detect in urine drug screens, at least amongst adolescents currently using substances.

The perceived benefits and risks associated with SM were higher in the dual-user group. Adolescents who used SM were more aware of medical, emotional, and addiction risks associated with SM than natural marijuana users. They also reported experiencing all the adverse events including agitation, tachycardia, paranoia, and mood swings, which have been reported in the earlier studies.^10,14^ Interestingly, SM users also reported more awareness of adverse experiences with natural marijuana than those who had never used SM. This enhanced risk awareness in adolescent SM users versus nonusers is somewhat surprising as adolescents tend to report less perceived risk of substances than adults.^20^ However, with the widespread advent of social media, today’s youth may feel well-informed about the potential risks of substance use.

Similar to risks, the benefits of SM were rated as higher in those using SM versus those adolescents who had never used it, suggesting that use may continue after initial experimentation as the perceived benefits appear to increase after use. Adolescents transitioning to SM use may be more predisposed to rate it more positively than those not transitioning to use; however, we did not collect ratings of the palatability of SM before and after its use, although dual users did not rate higher benefits of natural marijuana use than natural marijuana users. Despite more positive ratings of SM than those reporting never using SM, dual users rated higher benefits (quality, duration, and safety) of natural marijuana over SM, as well as higher risks of SM use when compared to natural marijuana use, with minimal difference in cost, raising the question of why use transitioned from natural to synthetic marijuana. Temperament or other personality factors (eg, novelty seeking, neuroticism)^27-29^ may be more predictive of the uptake of new substances, including synthetic substances, than the experience of having used that substance. Furthermore, comorbid externalizing symptoms or peer pressure might contribute to this transition.

These data signal that perceived risk does not motivate use in adolescents who have progressed to a substance use disorder, as perceived benefit is a much more impactful draw than perceived risks. Others have also reported that knowledge of the risks associated with substance use does not impact future use of that substance in adolescents.^30^ Therefore, treatment efforts focusing on emphasizing risks of ongoing use, at least health risks, may be of limited utility. Intervention programs may need to expand beyond risk awareness to curb the use of new synthetic products by adolescents and future study should focus on this.

This cross-sectional study has several limitations, including using a small, convenience sample of primarily white male adolescents receiving treatment for substance use disorders at a community adolescent substance use treatment program and inherent limitations (eg,—sexual orientation) of survey. Study findings must be replicated in other settings using a larger and more diverse sample. Furthermore, overall use of SM was relatively low compared to natural marijuana, and participants had to retrospectively recall their experiences with SM over the past 12 months.

Synthetic and designer products pose a significant risk to public health. Despite decreases in use of synthetic or designer products over the last decade, these substances will remain among adolescents’ evolving and diverse selection of substances.^26^ Adolescents with elevated levels of externalizing symptoms and a peer group experimenting with novel substances are at increased risk of transitioning to novel, risky substance use. Perceived risks do not appear to drive the uptake of novel substances in adolescents. The perceived benefits of using novel substances appear diverse, and merit further exploration as a better understanding of what motivates adolescents to use specific substances can be used to guide prevention and treatment efforts.

## Supplemental Material

sj-docx-1-sat-10.1177_29768357241254258 – Supplemental material for Synthetic Marijuana: Assessment of Usage, Motivation and Associated Risks in Adolescent Substance Users Supplemental material, sj-docx-1-sat-10.1177_29768357241254258 for Synthetic Marijuana: Assessment of Usage, Motivation and Associated Risks in Adolescent Substance Users by Raman Baweja, Sara Mills-Huffnagle, Amanda Jernigan, Nungshitombi Chongtham, Daniel Waschbusch and James G Waxmonsky in Substance Abuse: Research and Treatment
