# Supplementary material for: Synthetic Marijuana: Assessment of Usage, Motivation and Associated Risks in Adolescent Substance Users
Source: Subst Use. 2024 May 17;18:29768357241254258. doi: 10.1177/29768357241254258 (PMC11102655; doi:10.1177/29768357241254258)
Supplement: sj-docx-1-sat-10.1177_29768357241254258 – Supplemental material for Synthetic Marijuana: Assessment of Usage, Motivation and Associated Risks in Adolescent Substance Users [file sj-docx-1-sat-10.1177_29768357241254258.docx]

**Supplement**

***Substance Abuse Survey***

*Synthetic Marijuana is a man-made marijuana like chemical sprayed on plants that you usually smoke, swallow or inhale vapors. Names for it include: Space, Spice, K2, Spiggoty, Iggoty, or Fake Weed Natural Marijuana refers to marijuana that comes from a plant with no other chemicals added to it.*

*Have you ever heard of synthetic marijuana before?*

*Yes*

*No*

*I don’t know*

*I don’t want to answer*

*What is your gender?*

*Male*

*Female*

*I don’t know*

*I don’t want to answer*

*What is your race?*

*Caucasian (White)*

*African American*

*Hispanic*

*Mixed*

*Other*

*I don’t know*

*I don’t want to answer*

*If you selected “other” above, please describe your race: __________________________________*

*How many of your birth parents (mother/father) do you live with presently?*

*0*

*1*

*2*

*I don’t know*

*I don’t want to answer*

*What is the highest grade of school you have completed (8th, 9th, 10th, 11th, 12th, etc...)? __________________________________*

*Are you currently enrolled in school?*

*Yes*

*No*

*I don’t know*

*I don’t want to answer*

*How many days of school have you missed in the last three months of the school year for any reason: __________________________________ (enter a number)*

*How many days of school have you missed in the last three months of this school year due to problems with alcohol or drug use? __________________________________ (enter a number)*

*What is the highest level of school either one of your parents completed (select the best answer):*

*Less than high school - did not graduate*

*High school diploma or graduate equivalence degree (GED)*

*Attended college or trade school but did not graduate*

*Graduated college*

*Completed a degree after college (MBA, MD, PhD)*

*I don’t know*

*I don’t want to answer*

*What is your personal income per week (do not include money that comes from your parents like allowances)*

*None*

*$1-49*

*$50-100*

*$101-149*

*$150-200*

*$200 or more per week*

*I don’t know*

*I don’t want to answer*

*Do you currently have a job?*

*Yes*

*No*

*I don’t know*

*I don’t want to answer*

*Nights per week that you go out with friends:*

*None*

*1*

*2-3*

*4 or more*

*I don’t know*

*I don’t want to answer*

*Do any of your friends use synthetic marijuana?*

*None*

*Just a few*

*Most*

*All*

*I don’t know*

*I don’t want to answer*

*Do any of your friends use natural marijuana?*

*None*

*Just a few*

*Most*

*All*

*I don’t know*

*I don’t want to answer*

*Do any of your friends use other drugs?*

*None*

*Just a few*

*Most*

*All*

*I don’t know*

*I don’t want to answer*

*What is your sexual orientation?*

*Males*

*Females*

*Both*

*I don’t know*

*I don’t want to answer*

*Have you ever been arrested by the police?*

*Yes*

*No*

*I don’t know*

*I don’t want to answer*

*What is your current favorite drug to use (can also answer alcohol)?*

*__________________________________*

*How often have you used Synthetic Marijuana in the past 12 months?*

*None*

*1-2 times in the past 12 months*

*3-5 times in the past 12 months*

*6-9 times in the past 12 months*

*10-19 times in the past 12 months*

*20-39 times in the past 12 months*

*40 or more times in the past 12 months*

*I don’t know*

*I don’t want to answer*

*How many days have you used synthetic marijuana in the past month?*

*__________________________________ (enter a number)*

*How old were you when you first used synthetic marijuana?*

*__________________________________ (enter a number)*

*How did you use synthetic marijuana (check all that I did not use synthetic marijuana apply)?*

*I did not use synthetic marijuana*

*Smoke*

*Vapor*

*Eat or drink it*

*I don’t know*

*I don’t want to answer*

*In the year before entering treatment, did you have to take drug tests (either at home, work, school, for sports teams, etc.)?*

*Yes*

*No*

*I don’t know*

*I don’t want to answer*

*If you did have to take a drug test, how worried were you about failing one?*

*Not at all*

*A little*

*Some*

*A lot*

*It was one of my biggest worries*

*I don’t know*

*I don’t want to answer*

*How often have you used Natural (comes from a plant) Marijuana in the past 12 months?*

*None*

*1-2 times in the past 12 months*

*3-5 times in the past 12 months*

*6-9 times in the past 12 months*

*10-19 times in the past 12 months*

*20-39 times in the past 12 months*

*40 or more times in the past 12 months*

*I don’t know*

*I don’t want to answer*

*How many days have you used natural marijuana in the past month?*

*__________________________________ (enter a number)*

*How old were you when you first used natural marijuana?*

*__________________________________ (enter a number)*

*How did you use natural marijuana (check all that apply)?*

*I did not use natural marijuana*

*Smoke*

*Vapor*

*Eat or drink it*

*I don’t know*

*I don’t want to answer*

*Have you ever tried synthetic marijuana?*

*Yes*

*No*

*I don’t know*

*I don’t want to answer*

*If yes, did you use synthetic marijuana with friends or by yourself? __________________________________*

*If yes, how did you get the synthetic marijuana? __________________________________*

*If yes, why did you decide to try synthetic marijuana? __________________________________________*

*How did the synthetic marijuana make you feel? __________________________________________*

*When you use synthetic marijuana, why do you use (please check all that applies) i*

*To avoid being detected on drug tests*

*I like how it makes me feel*

*It’s cheap*

*It’s easy to get*

*It’s safe - no bad side effects*

*So I don’t go into withdrawal from other drugs*

*It’s popular right now*

*To improve the high of other drugs when I mix them together*

*I am addicted to it - can’t quit, even if I try*

*My friends made me use it*

*Other*

*I don’t know*

*I don’t want to answer*

*If you selected that you use synthetic marijuana because you like how it makes you feel, please tell us how it makes you feel: __________________________________________*

*If you selected “other” above, please describe why you use synthetic marijuana: __________________________________________*

*Out of the reasons you selected about for using synthetic marijuana, what is the main reason you used synthetic marijuana (please pick one reason)? __________________________________________*

*Please rate your experience with synthetic marijuana on a 1 to 10 scale:*

|  | *1* | *2* | *3* | *4* | *5* | *6* | *7* | *8* | *9* | *10* |
| --- | --- | --- | --- | --- | --- | --- | --- | --- | --- | --- |
| *How good the high is (1 is very poor, 10 is great)* |  |  |  |  |  |  |  |  |  |  |
| *How long the high lasts (1 very short, 10 very long)* |  |  |  |  |  |  |  |  |  |  |
| *How fast it takes to feel effects (1 very slow, 10 very fast)* |  |  |  |  |  |  |  |  |  |  |
| *Value for my money (1 terrible value, 10 great value)* |  |  |  |  |  |  |  |  |  |  |
| *Safest (1 very safe, 10 very dangerous)* |  |  |  |  |  |  |  |  |  |  |
| *Addiction risk (1 no risk of addition, 10 great risk of addiction)* |  |  |  |  |  |  |  |  |  |  |
| *Hangover effects (how you feel after it has worn off: 1 none, 10 severe)* |  |  |  |  |  |  |  |  |  |  |
| *Risk of seizures (1 no risk, 10 big risk)* |  |  |  |  |  |  |  |  |  |  |
| *Risk of feeling paranoid (1 no risk, 10 big risk)* |  |  |  |  |  |  |  |  |  |  |
| *Risk of becoming aggressive (1 no risk, 10 big risk)* |  |  |  |  |  |  |  |  |  |  |
| *Risk of breathing problems (1 no risk, 10 big risk)* |  |  |  |  |  |  |  |  |  |  |
| *Risk of mood wings (1 no risk, 10 big risk)* |  |  |  |  |  |  |  |  |  |  |

*Natural Marijuana Please rate your experience with natural marijuana on a 1 to 10 scale:*

|  | *1* | *2* | *3* | *4* | *5* | *6* | *7* | *8* | *9* | *10* |
| --- | --- | --- | --- | --- | --- | --- | --- | --- | --- | --- |
| *How good the high is (1 is very poor, 10 is great)* |  |  |  |  |  |  |  |  |  |  |
| *How long the high lasts (1 very short, 10 very long)* |  |  |  |  |  |  |  |  |  |  |
| *How fast it takes to feel effects (1 very slow, 10 very fast)* |  |  |  |  |  |  |  |  |  |  |
| *Value for my money (1 terrible value, 10 great value)* |  |  |  |  |  |  |  |  |  |  |
| *Safest (1 very safe, 10 very dangerous)* |  |  |  |  |  |  |  |  |  |  |
| *Addiction risk (1 no risk of addition, 10 great risk of addiction)* |  |  |  |  |  |  |  |  |  |  |
| *Hangover effects (how you feel after it has worn off: 1 none, 10 severe)* |  |  |  |  |  |  |  |  |  |  |
| *Risk of seizures (1 no risk, 10 big risk)* |  |  |  |  |  |  |  |  |  |  |
| *Risk of feeling paranoid (1 no risk, 10 big risk)* |  |  |  |  |  |  |  |  |  |  |
| *Risk of becoming aggressive (1 no risk, 10 big risk)* |  |  |  |  |  |  |  |  |  |  |
| *Risk of breathing problems (1 no risk, 10 big risk)* |  |  |  |  |  |  |  |  |  |  |
| *Risk of mood wings (1 no risk, 10 big risk)* |  |  |  |  |  |  |  |  |  |  |

*Have you ever had to go to the hospital or to get medical treatment because of problems with synthetic marijuana (bad high, withdrawal side effects, etc.)?*

*Yes*

*No*

*I don’t know*

*I don’t want to answer*

*Please describe the worst experience you have had with synthetic marijuana:*

*__________________________________________*

*Have you ever been diagnosed with a mental health or emotional disorder (depression, anxiety, ADHD, Bipolar Disorder, Schizophrenia, etc.)*

*Yes*

*No*

*I don’t know*

*I don’t want to answer*

*If yes, please tell us which disorder(s):*

*Have you ever been treated for a mental health or emotional disorder (depression, anxiety, ADHD, Bipolar Disorder, Schizophrenia, etc.) with medication?*

*Yes*

*No*

*I don’t know*

*I don’t want to answer*

*If yes, please tell us which disorder(s): __________________________________*

*Have you ever been in the hospital because of a mental health or emotional disorder (depression, anxiety, ADHD, Bipolar Disorder, Schizophrenia, etc.)?*

*Yes*

*No*

*I don’t know*

*I don’t want to answer*

*Does anybody else in your family have a problem with drugs or alcohol?*

*Yes*

*No*

*I don’t know*

*I don’t want to answer*

*How many days have you used nicotine (cigarettes, chewing tobacco, cigars) in the last month? __________________________________ (enter a number)*

*How many days have you used alcohol in the last month?*

*__________________________________ (enter a number)*

*Have you used any other drugs besides marijuana?*

*Yes*

*No*

*I don’t know*

*I don’t want to answer*

*How many days have you used cocaine in the past month?*

*__________________________________ (enter a number)*

*How old were you when you first used cocaine?*

*__________________________________ (enter a number)*

*How many days have you used narcotics (heroin, morphine, codeine, demoral, methadone, percodan, Darvon, oxycontin, etc.) in the past month?*

*__________________________________ (enter a number)*

*How old were you when you first used narcotics?*

*__________________________________ (enter a number)*

*How many days have you used amphetamine or methamphetamine in the past month? __________________________________ (enter a number)*

*How old were you when you first used amphetamine or methamphetamine? __________________________________ (enter a number)*

*How many days have you used ADHD medications (Ritalin, Adderall, Concerta, Vyvanse, etc.) in the past month for something other than treating your own ADHD?*

*__________________________________ (enter a number)*

*How old were you when you first used ADHD medications that weren’t prescribed for you? __________________________________ (enter a number)*

*How many days have you used benzodiazepines (valium, Librium, serax, Ativan, dalmane, Xanax, etc.) in the past month?*

*__________________________________ (enter a number)*

*How old were you when you first used benzodiazepines?*

*__________________________________ (enter a number)*

*How many days have you used hallucinogens (LSD, ecstasy, mescaline, mushrooms, etc.) in the past month? __________________________________ (enter a number)*

*How old were you when you first used hallucinogens?*

*__________________________________ (enter a number)*

*How many days have you used inhalants in the past month?*

*__________________________________ (enter a number)*

*How old were you when you first used inhalants?*

*__________________________________ (enter a number)*
